# Propene epoxidation with molecular oxygen: Advancements from nanoparticle to single‐atom catalysts

**DOI:** 10.1002/smo.20240025

**Published:** 2024-11-24

**Authors:** Qiuming He, Dong Lin, Defu Yin, Chaohe Yang, De Chen, Xiang Feng

**Affiliations:** ^1^ State Key Laboratory of Heavy Oil Processing China University of Petroleum Qingdao China; ^2^ Max Planck‐ Cardiff Centre on the Fundamentals of Heterogeneous Catalysis FUNCAT Cardiff Catalysis Institute School of Chemistry Cardiff University Cardiff UK; ^3^ Department of Chemical Engineering Norwegian University of Science and Technology Trondheim Norway

**Keywords:** epoxidation mechanism, molecular oxygen, nanoparticle catalyst, propene epoxidation, single‐atom catalyst

## Abstract

Propylene oxide plays a pivotal role as an organic synthesis intermediate, boasting extensive downstream applications and promising market prospects. Propene epoxidation via molecular oxygen has garnered considerable attention due to its cost‐effectiveness, environmental friendliness, ease of operation, and straightforward product separation. This paper provides an in‐depth exploration of recent advancements, ranging from nanoparticle to Single‐atom catalysts (SACs), in the context of propene epoxidation using molecular oxygen. Conventional nanoparticle catalysts, including those based on Ag, Cu, and other metals, are examined with regard to their contributions to support effects, electron effects, or crystal‐plane effects within the mechanistic investigation. Furthermore, emerging SACs (specifically Mo, Cu, and Co) are discussed in terms of synthesis strategies, characterization methods, and mechanism studies. This comprehensive review sheds new light on design strategies, relevant characterizations, and thorough mechanism investigations aimed at fostering the development of efficient catalysts, thereby expediting progress in the industrial implementation of propene epoxidation.

## Graphical abstract

This paper offers a comprehensive review about propene epoxidation using molecular oxygen, focusing on catalysts that range from nanoparticles (Ag, Cu, and other metals) to single atoms (specifically Mo, Cu, and Co). It reviews nanoparticle catalysts by exploring support, electron, and plane effects within mechanistic investigations. Additionally, it discusses emerging single‐atom catalysts, covering synthesis strategies, characterizations, and mechanisms.

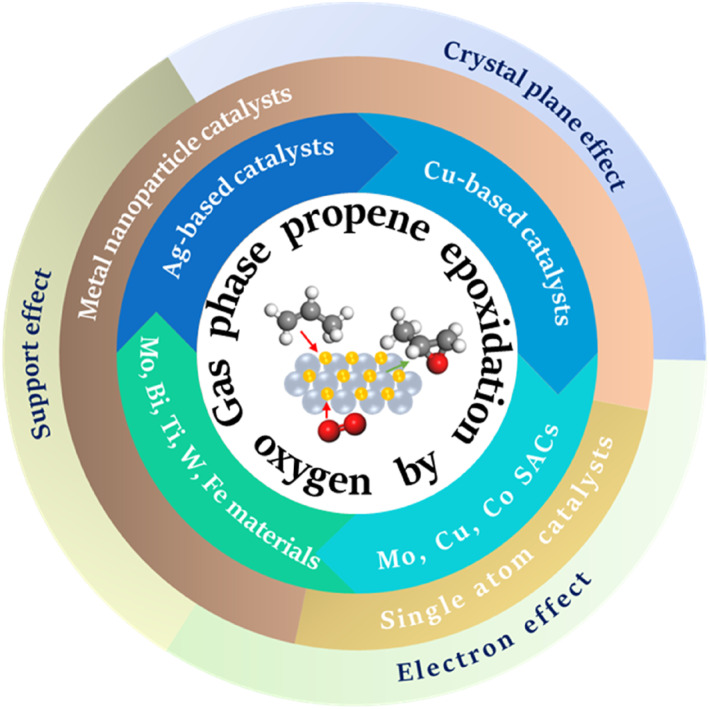

## 1 INTRODUCTION

Propylene oxide (PO) is a vital organic synthetic material and a versatile low‐boiling‐point solvent, making it a crucial downstream product of propylene. PO is employed in the production of various substances, including propylene glycol, glycerol, polyester resins, foam and surfactants.^[1]^ Of these, polyether polyols are the primary raw material for polyurethane foam, propylene glycols are used as raw material for the production of unsaturated polyester resins applied in the textile and construction industries, and propylene glycol ethers serve as solvents in paints, inks and many other related applications.^[2]^

**FIGURE 1:**
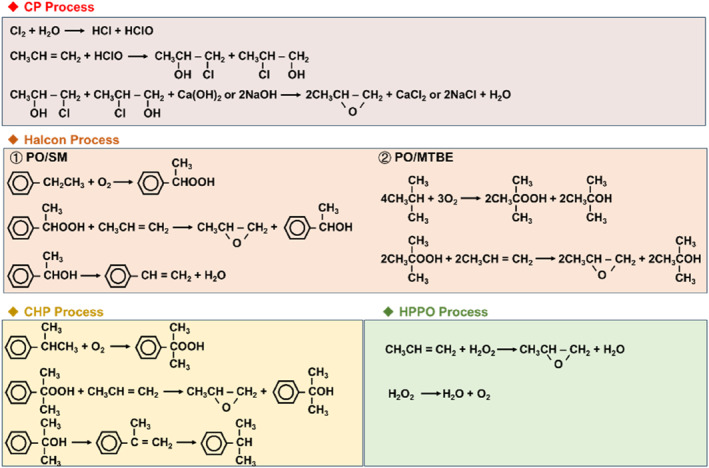
Reactions involved in the industrial production of Propylene oxide (PO).

The industrial production processes for PO consist of the chlorohydrin process (CP), co‐oxidation process (Halcon), cumene hydrogen peroxidation process (CHPPO), and the peroxide oxidation method (HPPO), as shown in Figure 1. The CP process, recognized for its maturity and simplicity, has low feedstock purity demands and excellent product selectivity. Nevertheless, it is being phased out of the market due to notable environmental pollution and equipment corrosion concerns.^[3]^ The Halcon process is environmentally friendly and causes less equipment corrosion. However, it requires high purity gas feedstock and produces significant quantities of tert‐butyl alcohol or styrene, which may encounter market limitation.^[4]^

The CHPPO process is a modification of the Halcon process but doesn't produce any by‐products. It allows feedstock recycling without needing extra anti‐corrosion equipment, though it comes with high operational expenses and necessitates large supporting units. The HPPO process, an emerging green approach with environmentally friendly products, is significantly impacted by the quality of the H_2_O_2_ feedstock.^[5]^ Additionally, direct propene epoxidation with H_2_ and O_2_ is gradually emerging. This process eliminates the need for H_2_O_2_ extraction and continuous distillation, thereby significantly reducing energy consumption. However, the reported yield of propylene oxide is lower than that of the ex‐situ HPPO process. Since Haruta and his colleagues first reported it,^[6]^ the one‐step alternative method via in‐situ H_2_O_2_ synthesis has been a major focus area and has the potential to offer higher efficiency compared to alternative processes. Notably, gold nanoparticles deposited on titanosilicalite have been reported to be highly efficient for in‐situ propene epoxidation.

Direct propene epoxidation with oxygen is widely recognized as the cleanest and most atomically efficient approach for PO production, earning recognition as the “holy grail” of propene epoxidation systems. It has attracted widespread interest from both academia and industry. However, challenges such as low PO selectivity, limited propylene conversion, and unstable catalysts hinder the industrial implementation of this process. Consequently, extensive research has been dedicated to overcoming these obstacles and advancing the direct propene epoxidation to PO using oxygen.[^7^, ^8^] For industrially relevant direct propene epoxidation with oxygen, the catalyst should meet the following basic requirements^[9]^: (1) high PO selectivity to improve the atom economy of the reaction process (the higher, the better, with at least >50%); (2) reasonable single‐pass conversion of propylene in the cyclic reaction (at least >10%); and (3) good catalyst stability, which is beneficial for reactor design and process economics. Additionally, for safety reasons, the concentration of propylene and O₂ in the feed gas should be carefully controlled (O_2_ concentration limit: 8.2%).

**FIGURE 2:**
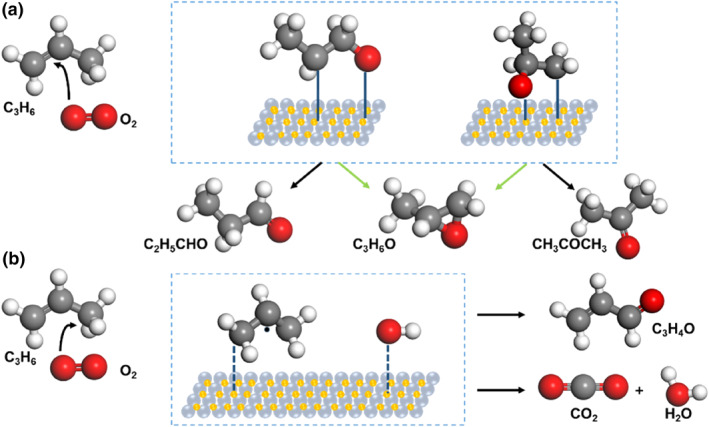
Reaction pathways for the oxidation of propylene with O_2_: (a) Selective oxidation. (b) Allylic hydrogen extraction. The grey, red, white ball represents C, O, H atom, respectively.

The allyl group in propylene is known to be more reactive than the C=C double bond, leading to complete oxidation rather than partial oxidation.[^8^, ^10^] Figure 2 illustrates the reaction pathways for propene oxidation with O_2_, where partial oxidation products can subsequently undergo further oxidation to produce CO_2_ through adsorbed oxygen. Both linear and branched configurations of oxametallacycle (OMC) can form when oxygen attacks the C=C double bond. Additionally, the number of metal atoms forming the ring is a determining factor for the oxidation pathways, regardless of OMC configuration. The regulation of adsorbed oxygen characteristics and the activity of catalyst sites is vital in determining the pathway to epoxidation products.

**FIGURE 3:**
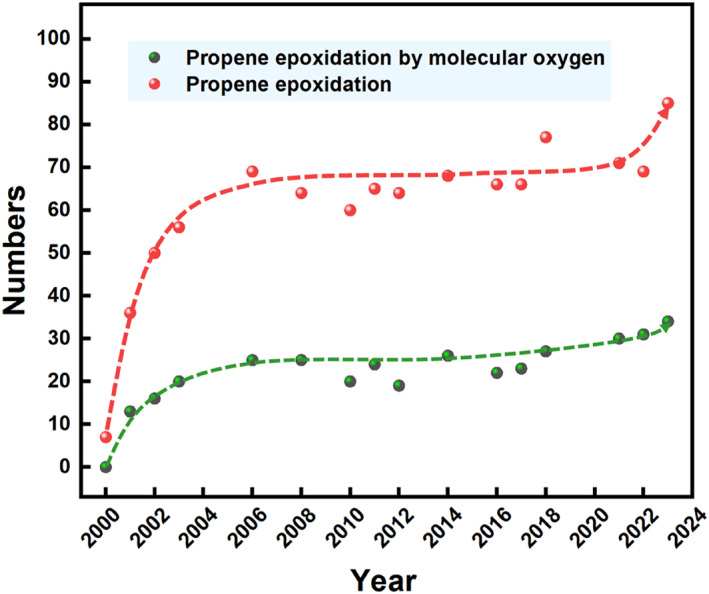
Trends in the number of publications from 2000 to 2023. The former of which was searched by those of “propylene epoxidation by oxygen” and “propylene oxide” and the latter of which by terms of “propylene epoxidation” and “propylene oxide” from Web of Science.

**FIGURE 4:**
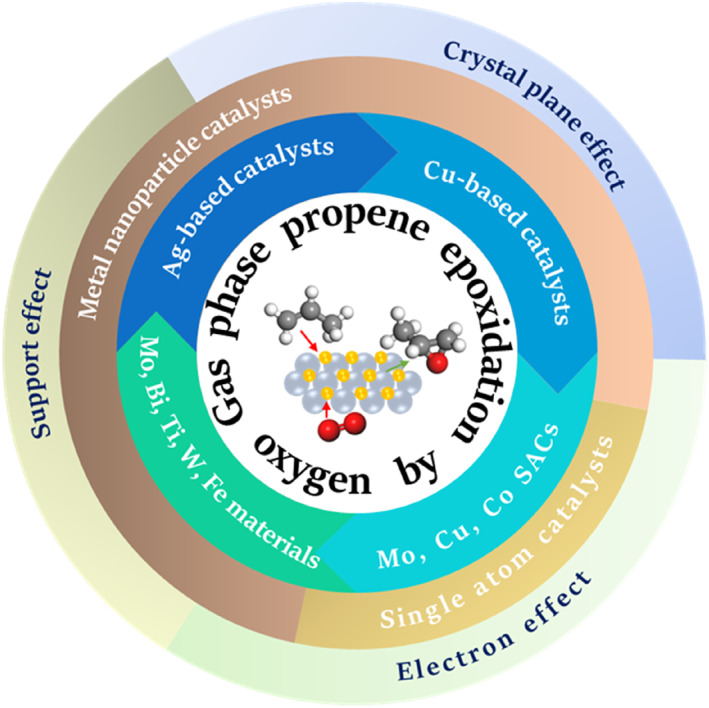
Schematic diagram of catalysts from nanoparticle to single atom for propene epoxidation.

Figure 3 depicts the yearly distribution trend of literature in the field of propene epoxidation and propene epoxidation using molecular oxygen over the past 3 decades. The graph shows that the number of publications on these topics has reached a higher plateau in the last 30 years after a rapid growth and is still on the rise in the recent 5 years. While several reviews on the gas‐phase propene epoxidation have been published,[^7^, ^8^, ^10^, ^11^, ^12^, ^13^] most of their review catalytic systems involving the gas‐phase epoxidation reaction of propylene with reductive gases such as H_2_, NO, N_2_O and CO, rather than a more environment‐friendly propene epoxidation with only oxygen as the oxidant. Comprehensive research on the current status of catalytic systems for the propene epoxidation solely using oxygen is lacking. In this review, we summarize recent progress in transitioning from metal nanoparticle catalysts to Single‐atom catalysts (SACs) for the direct propene epoxidation with oxygen. Our focus lies in understanding the influence of catalyst structure and composition on catalytic performance, as well as exploring the impact of support materials, electron interactions, and crystal facets on the propene epoxidation with oxygen (Figure 4). This review illuminates innovative design strategies, pertinent characterizations, and in‐depth mechanistic studies with the goal of advancing the creation of efficient catalysts, thus accelerating the advancements in the industrial adoption of propene epoxidation by oxygen.

## 2 METAL NANOPARTICLE CATALYSTS

In recent years, effort has been carried out on the crucial factors that govern catalytic performance, which include size, composition, valence of the active metal components, crystal plane, and the surface area of the supports. These factors alter the electronic and/or geometrical structure of the catalyst and ultimately its reactivity and selectivity.[^14^, ^15^, ^16^, ^17^, ^18^] While inert metal like gold (Au) demonstrates excellent epoxidation performance in hydrogen (H_2_) conditions, there are no reports of Au catalysts capable of catalyzing the direct propene epoxidation to PO using oxygen as the oxidizing agent. In contrast, copper (Cu) and silver (Ag) catalysts exhibit superior activity and selectivity in the direct propene epoxidation.

### 2.1 Ag‐based catalysts

#### 2.1.1 Support effect

The choice of a supporting material plays a crucial role in regulating the chemical composition, exposing active sites, and fostering synergy at the metal‐support interface. In fact, the support effect and metal particle size are closely connected. Early studies found that CaCO_3_ emerges as an effective support for Ag‐based catalysts in catalyzing the propene epoxidation. Notably, support materials with low symmetry or irregular shapes, such as the scale of calcite decahedron, can notably enhance catalyst performance. Additionally, the introduction of potassium (K) to the Ag/CaCO_3_ catalyst led to a significant improvement in selectivity of PO. Researchers have primarily focused on the impact of supports and promoters[^19^, ^20^, ^21^] as the morphology of the supports and the addition of promoters can influence the size and dispersion of Ag nanoparticles.

**FIGURE 5:**
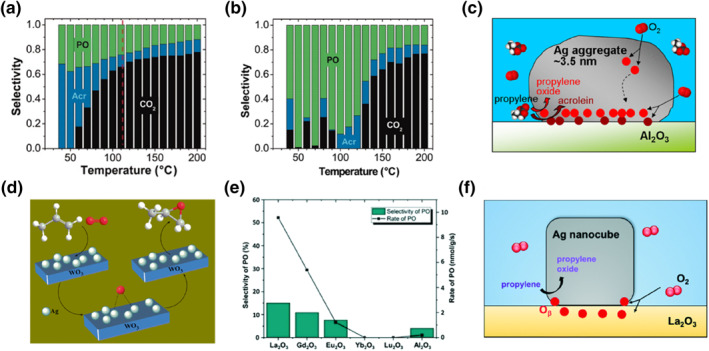
(a) Selectivity of propylene, acrolein, and CO_2_ versus temperature on Ag_3_ clusters, (b) Selectivity of propylene, acrolein, and CO_2_ versus temperature on nanoparticle aggregates, (c) Schematic illustration of various reactions that occur on the supported Ag aggregate, (d) Schematic diagram for propene epoxidation on Ag/WO_3_ catalyst, (e) Propylene oxide (PO) selectivity and productivity rates exhibited by Ln_2_O_3_ (La, Gd, Eu, Yb, Lu) and Al_2_O_3_‐supported Ag nanospheres, (f) Hypothetical reaction mechanism of selective propene epoxidation over the La_2_O_3_‐supported Ag nanocube catalyst. Reproduced with permission from Ref. [22]. Copyright 2010, American Association for the Advancement of Science. Reproduced with permission from Ref. [23], [24]. Copyright 2014, American Chemical Society. Reproduced with permission from Ref. [25]. Copyright 2019, Royal Society of Chemistry.

In their exploration of Ag single‐metal catalysts, Lei et al.^[22]^ discovered that Ag_3_ clusters and ∼3.5 nm Ag nanoparticles supported on alumina exhibited remarkable activity at low temperatures. These catalysts effectively promoted the propene epoxidation while producing negligible amounts of carbon dioxide (see Figures 5a,b). Notably, the formation rate of PO molecules approached approximately 1 s^−1^ per surface silver atom at 110°C. They delved into the catalytic mechanism of alumina‐supported Ag trimers.^[23]^ In cases involving Ag aggregates, silver interfacial oxygen played a key role in PO formation, whereas alumina interfacial oxygen facilitated acrolein formation, as depicted in Figure 5c. The O_2_ dissociates on the interfacial site, resulting in one on the Ag cluster (O_intf, Ag_) and the other on the support (O_intf, s_) while still bound to the Ag cluster. In addition, the oxygen atoms in the product of O_2_ dissociation on a top site of the Ag cluster are referred to as O_top_. Their calculations show that O_intf, Ag_ sites are both thermodynamically and kinetically active for propene epoxidation. The non‐interfacial sites, such as O_intf, s_ or O_top_, are less active, but O_top_ sites can migrate to the interfacial sites to complement O_intf, Ag_ sites and react with propylene to form PO. These findings suggest that the interface between alumina and Ag aggregates serves as the reactive center, with the amorphous surface playing a significant role in facilitating the catalytic reaction of propene epoxidation that takes place on it. Similarly, Molina^[26]^ prepared silver nano‐catalysts ranging in size from 9 to 23 nm on amorphous alumina films. Their study revealed that smaller clusters were more inclined to produce acrolein, while the 23 nm particles were deemed to be more selective in the formation of PO.

Ag nanoparticle catalysts ranging from 2‐5 nm in size, supported on WO_3_ nanorods were prepared using surfactant cetyltrimethylammonium bromide (CTAB), capping agent polyvinylpyrrolidone (PVP), and hydrazine.^[24]^ The Ag/WO_3_ catalyst, with 4.8 wt% Ag loading under reaction conditions of 2 MPa and 250°C, achieved a propylene conversion of 15.5% and a remarkable PO selectivity of 83%, resulting in a PO yield of 6.1 × 10^−2^ mol·g_cat_^−1^ h^−1^. In order to understand whether WO_3_ nanorods are necessary in the propene epoxidation, the researchers also prepared several other metal‐oxide supports and assessed their catalytic activity. Catalysts loaded with Ag, such as those supported by Cr_2_O_3_ and MnO_3_, exhibited lower activity compared to the Ag/WO_3_ nanostructured catalysts. Additionally, WO_3_‐loaded CuO particles showed approximately 4% propylene conversion and 60% PO selectivity but suffered deactivation after 3 h, likely due to sintering of the Cu particles. In contrast, a strong synergy was observed between the surface Ag nanoparticles and WO_3_ nanorods, indicating their effectiveness in enhancing catalytic performance. The rod structure of WO_3_, as depicted in Figure 5d, played a vital role in enhancing the uniform dispersion of ultrasmall metallic Ag nanoparticles, which, in turn, promotes the dissociation of molecular oxygen on the metallic Ag surface, leading to the formation of Ag_2_O. The WO_3_ support not only prevents coalescence and aggregation of Ag nanoparticles but also greatly facilitates the formation of PO.

Similarly, adopting titanium‐containing hexagonal mesoporous silica (Ti‐HMSn) with different Si/Ti ratios as a support for Ag particles controlled the size of Ag particles down to 6.8 nm, resulting in a notable increase in PO yield.^[27]^

Lanthanide oxides are well‐established for creating catalytically active adsorbed oxygen on various catalysts by surface oxygen vacancies, while maintaining the A‐type sesquioxide‐Ln_2_O_3_ structure.[^28^, ^29^, ^30^] A study by Yu et al.^[25]^ explored Ag nanoparticles supported by various metal oxides, investigating changes in the electronic structure at the interface between Ln_2_O_3_ and Ag nanoparticles. The screening tests of different Ag/Ln_2_O_3_ catalysts for propylene oxidation demonstrated that catalysts with La_2_O_3_‐supported Ag nanoparticles exhibited the highest activity in generating epoxidation products, as depicted in Figure 5e. To assess the impact of Ag morphology, they conducted propene epoxidation experiments using two different shapes of single‐metal silver, namely Ag nanospheres and Ag nanocubes. Remarkably, Ag nanocubes exhibited twice the selectivity for PO compared to Ag nanospheres under the same support and conditions at 250°C. Furthermore, the study indicated that the higher prevalence of Ag (100) facets in Ag nanocubes significantly facilitated the formation of PO compared to Ag nanospheres. Ag nanocubes supported by La_2_O_3_ led to substantial improvements in propylene conversion and PO selectivity, increasing by 11.6% and 51%, respectively, at 270°C. Notably, even in the absence of hydrogen (H_2_) and other additives, Ag/La_2_O_3_ displayed superior activity at low temperatures and pressures. The researchers concluded that adjusting the surface morphology of the two activated oxygen atoms on the Ag surface is critical for enhancing PO selectivity. As depicted in Figure 5f, molecular oxygen demonstrated a propensity to dissociate on the polar La_2_O_3_ surface, migrating to the Ag (100) surface near the Ag‐La_2_O_3_ interface to form active O_*β*_ species. Electrophilic atomic oxygen adsorbed on the Ag surface interacted with propylene to produce oxametallacycle intermediates, ultimately yielding PO. The Ag/La_2_O_3_ interface was identified as the active oxygen site for PO formation. Furthermore, Pulido and colleagues^[31]^ reported that Ag (100) exhibited higher activity in PO formation compared to Ag (111).

The correlation between the support effect and metal particle size is significant. Specifically, support materials with low symmetry or irregular shapes can notably enhance catalyst performance. The morphology of supports and the addition of promoters affect the size and dispersion of Ag nanoparticles. CaCO_3_ proves to be an effective support for Ag‐based catalysts in propene epoxidation. Furthermore, incorporating potassium (K) into the Ag/CaCO_3_ catalyst markedly improves PO selectivity. The interface between alumina and Ag aggregates acts as the reactive center, with the amorphous surface playing a crucial role in facilitating the catalytic reaction of propene epoxidation occurring on it. The rod structure of WO_3_ is essential for enhancing the uniform dispersion of ultrasmall metallic Ag nanoparticles, thereby promoting the dissociation of molecular oxygen on the metallic Ag surface. The WO_3_ support not only prevents the coalescence and aggregation of Ag nanoparticles but also significantly aids in PO formation. Lanthanide oxides are well‐recognized for generating catalytically active adsorbed oxygen on various catalysts via surface oxygen vacancies, while maintaining the A‐type sesquioxide‐Ln_2_O_3_ structure.

#### 2.1.2 Electron effect

The electronic structure of the active metal significantly impacts catalytic activity. Researchers have modified the microenvironment of active metals by introducing heteroatoms. For example, Takahashi et al.^[32]^ explored the propene epoxidation of Ag‐based catalysts modified with transition metals (Mn, Fe, Co, Ni). Among these catalysts, the one containing 33 mol% Ni exhibited the most notable improvement in epoxidation activity and selectivity. Operating at 0.3 MPa pressure and 170°C, it achieved a 6.5% propylene conversion and a 9.8% PO selectivity.

**FIGURE 6:**
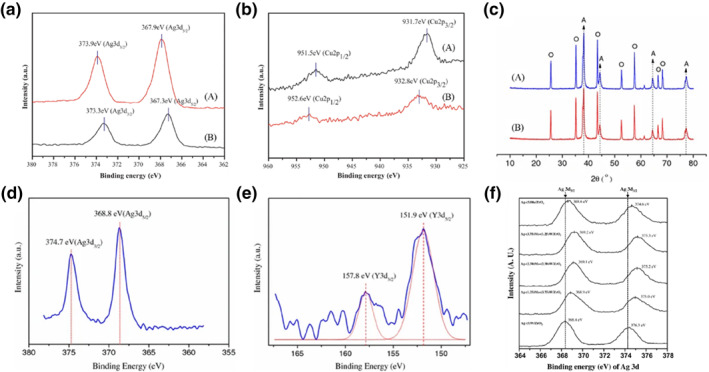
(a) Ag3d X‐ray Photoelectron Spectroscopy (XPS) spectra of Ag_95_‐Cu_5_/BaCO_3_ (A) and Ag/BaCO_3_ (B) catalysts, (b) Cu2p XPS spectra of Ag_95_‐Cu_5_/BaCO_3_ (A) and Ag/BaCO_3_ (B) catalysts, (c) XRD pattern of 20%Ag‐0.1%K_2_O/α‐Al_2_O_3_ (A) and 20%Ag‐0.1%Y_2_O_3_‐0.1%K_2_O/α‐Al_2_O_3_ (B) catalysts (O, *α*‐Al_2_O_3_;A, Ag), (d) Ag3d XPS spectra of 20%Ag‐0.1%Y_2_O_3_‐0.1%K_2_O/α‐Al_2_O_3_ catalyst, (e) Y3d XPS spectra of 20%Ag‐0.1%Y_2_O_3_‐0.1%K_2_O/α‐Al_2_O_3_ catalyst, (f) XPS spectra of Ag 3d_5/2_ and Ag 3d_3/2_ of Ag‐(*x*)Mo‐(5−*x*)W/ZrO_2_ (*x* = 5, 3.75, 2.50, 1.25, and 0) catalysts. Reproduced with permission from Ref. [33]. Copyright 2012, Elsevier. Reproduced with permission from Ref.[34]. Copyright 2007, Springer. Reproduced with permission from Ref.[35]. Copyright 2017, Elsevier.

Expanding catalysts from single‐metal to bimetallic catalysts is key to achieving efficient chemical processes. Studies have consistently indicated that bimetallic or multi‐metallic catalytic systems tend to exhibit better catalytic performance for propene epoxidation compared to single‐metallic systems. An Ag‐Cu/BaCO_3_ bimetallic catalyst, prepared by the surfactant‐protected colloidal method, was investigated for the propene epoxidation by molecular oxygen.^[33]^ This bimetallic catalyst displayed superior catalytic performance for molecular oxygen epoxidation in comparison to other supported Ag‐based bimetallic catalysts and single‐metallic catalysts. The catalytic performance of various BaCO_3_‐supported bimetallic catalysts for propene epoxidation by molecular oxygen is detailed in Tables 1 and 2. Particularly, at 200°C, the Ag_95_‐Cu_5_/BaCO_3_ bimetallic catalysts achieved the highest PO selectivity at 55.1% and a propylene conversion of 3.6%. Furthermore, as illustrated in Figure 6a,b, X‐ray Photoelectron Spectroscopy (XPS) results indicated that the presence of Cu had the effect of extracting electrons from the vicinity of Ag, rendering Ag positively charged. This positive charge proved conducive to generating more active sites, facilitating the absorption of electrophilic oxygen, and ultimately enhancing the selectivity of PO.

**TABLE 1:** Catalytic performance of the supported Ag‐based bimetallic catalysts.^[33]^

Catalysts	C_3_H_6_ conversion	PO selectivity	CO_2_ selectivity
Ag‐Fe/BaCO_3_	5.8%	3.2%	96.8%
Ag‐Co/BaCO_3_	8.1%	4.1%	95.9%
Ag‐Ni/BaCO_3_	6.5%	1.6%	98.4%
Ag‐Mn/BaCO_3_	9.2%	2.5%	97.5%
Ag‐Mo/BaCO_3_	1.6%	4.2%	95.8%
Ag‐V/BaCO_3_	1.4%	3.6%	96.4%
Ag‐Zn/BaCO_3_	2.6%	3.1%	96.9%
Ag‐Cu/BaCO_3_	3.6%	55.1%	44.9%
Ag/BaCO_3_	2.4%	10.2%	89.8%
Cu/BaCO_3_	0.9%	5.5%	94.5%

**TABLE 2:** Effect of molar ratio of Ag/Cu on the catalytic performance of Ag‐Cu/BaCO_3_ catalysts.^[33]^

Catalysts	C_3_H_6_ conversion	PO selectivity	CO_2_ selectivity
Ag_80_‐Cu_20_	6.4%	15.1%	84.9%
Ag_90_‐Cu_10_	4.6%	40.4%	59.6%
Ag_95_‐Cu_5_	3.6%	55.1%	44.9%
Ag_98_‐Cu_2_	3.3%	36.2%	63.8%
Ag_99_‐Cu_1_	3%	24.3%	75.7%

The CuO_*x*_, AgO_*x*_, and AgCu_*x*_ bimetallic catalysts were also prepared and their catalytic performance and active sites for propene epoxidation were investigated.^[36]^ The study revealed that propylene conversion increased significantly with an increasing Cu/Ag molar ratio and then gradually decreased when exploring the catalytic performance of AgCu_*x*_ bimetallic catalysts. The PO selectivity exhibited a sharp increase, reaching a maximum of 27.4% when Cu/Ag = 1/8 and subsequently decreased. The catalytic performance of AgCu_*x*_ catalysts was further enhanced when a support was employed. Specifically, when *α*‐Al_2_O_3_ support was modified with Cs_2_O, the propylene conversion of Ag_8_Cu_1_/Cs_2_O/α‐Al_2_O_3_ reached 5.5%, with a PO selectivity of 48.5%. The modification of the support with alkaline oxides effectively inhibited the conversion of PO to carbon dioxide and water.

The Ag/α‐Al_2_O_3_ catalysts modified with rare earth metal oxides (Y_2_O_3_) and alkali metal oxides (K_2_O) for the molecular oxygen epoxidation of propylene were prepared by Lu.^[34]^ Among these catalysts, the 20% Ag‐0.1% Y_2_O_3_‐0.1% K_2_O/α‐Al_2_O_3_ exhibited a propylene conversion of 4% and a PO selectivity of 46.8% at 245°C. The Ag/α‐Al_2_O_3_ catalysts modified with Y_2_O_3_ and K_2_O displayed commendable catalytic performance for the gas‐phase propene epoxidation. As shown in Figure 6c, the results indicated that the addition of 0.1 wt% Y_2_O_3_ reduced the Ag crystallites from 17.4 to 15.7 nm, suggesting that a small amount of Y_2_O_3_ effectively inhibited the agglomeration of Ag crystallites and acted as a structural promoter. Additionally, Figure 6d displayed higher binding energies of Ag 3d5/2 and Ag 3d3/2 in comparison to the binding energies of metallic Ag 3d5/2 (367.9 eV) and Ag 3d3/2 (373.9 eV). Figure 6e further demonstrated that the binding energies of Y 3d5/2 and Y 3d3/2 were lower than the binding energies of pure Y_2_O_3_ powder (Y 3d5/2156.4 eV, Y 3d3/2158.2 eV). These results suggested a transmission of electrons between Y_2_O_3_ and Ag particles, highlighting the dual role of Y_2_O_3_ as both a structural and electronic promoter.

Lu et al.[^37^, ^38^, ^39^] demonstrated that ZrO_2_ is a suitable support for Ag catalysts, significantly enhancing PO selectivity and offering excellent stability. The addition of MoO_3_ proved beneficial in further improving the catalyst's PO selectivity, with the slurry method yielding the best performance. The introduction of Mo^6+^ ions in the catalyst enabled the capture of electrons from Ag, resulting in a positive charge on the Ag surface. This, in turn, weakened the effective charge of absorbed oxygen on the Ag site, rendering the absorbed oxygen more electrophilic and thus facilitating the formation of PO. The support's role is akin to that of a promoter, enhancing the electrophilicity of the absorbed oxygen and influencing the catalyst's particle size and pore dimensions. Subsequently, the authors found that the addition of alkali metal chlorides like CaCl_2_, NaCl, and CsCl facilitated the propene epoxidation reaction on Ag‐catalyzed systems.

To delve into the effect of molybdenum content in detail on the catalytic activity of direct propene epoxidation to PO by molecular oxygen, a series of Ag‐(*x*)Mo‐(5−*x*)W/ZrO_2_ catalysts were prepared.^[35]^ The results revealed that all Ag‐(*x*)Mo‐(5−*x*)W/ZrO_2_ (*x* = 5, 3.75, 2.50, 1.25, and 0) catalysts exhibited higher selectivity for PO than the Ag/ZrO_2_ catalyst. The Ag/ZrO_2_ catalyst achieved a conversion of 48.4% and a PO selectivity of 2.3%, but displayed stable catalytic performance throughout the reaction. Particularly, the Ag‐(3.75)Mo‐(1.25)W/ZrO_2_ catalyst exhibited outstanding performance, yielding a 13% conversion of propylene and a 60% selectivity for PO. The molybdenum content in Ag‐(*x*)Mo‐(5−*x*)W/ZrO_2_ catalysts had a substantial impact on the selectivity for PO. The trend in selectivity for PO generally corresponded to the change in Ag 3d binding energy, as depicted in Figure 6f. X‐ray Photoelectron Spectroscopy analysis indicated that the addition of a promoter led to a higher binding energy of Ag due to electron transfer from the Ag atoms to the promoter. This process, connected to increased electrophilicity of absorbed oxygen at the Ag active site, decreased nucleophilicity, ultimately inhibiting the complete oxidation of propylene. This is consistent with previous reports indicating that transition metals, alkali (earth) metals, and other promoters can decrease the nucleophilicity of absorbed oxygen, leading to stronger electrophilicity of the absorbed oxygen, which inhibits the complete oxidation of the absorbed oxygen via interaction with allyl hydrogen in propylene. This subsequently increases the potential for the reaction between olefin carbon and the absorbed oxygen.

The authors also explored the effect of the pH of the ZrO_2_ support on catalytic performance, observing an increase in the selectivity for PO with a higher ratio of acidity to alkalinity on the catalyst surface. Among the tested catalysts, the Ag‐(Mo‐W)/ZrO_2_(pH = 10) catalyst exhibited the highest catalytic performance, achieving a PO selectivity of 68% and a propylene conversion of 13%.^[40]^

The chloride anion changes the electronic properties of the catalyst surface and can induce nearby oxygen atoms absorbed on Ag to be more electrophilic.^[41]^ Zhang et al.^[42]^ synthesized Ag‐CuCl_2_ catalysts, with an initial 1.3% propylene conversion, achieving remarkably high initial PO selectivity of 71.2%. Over time, the PO selectivity decreased to 13.9%, while propylene conversion increased to 3.2% after 500 min. The authors suggested that the close contact interface between Ag and CuCl_2_ can form epoxidation‐active molecular oxygen species, which serve as the active centers for the propene epoxidation reaction. Furthermore, the Ag‐Cu‐Cl catalyst was designed to investigate in detail the influence of Cu and Cl element content on propene epoxidation performance.^[43]^ The Ag‐Cu‐Cl/BaCO_3_ catalyst exhibited optimal performance when Cu and Cl loadings were at 0.036 wt% and 0.060 wt%, respectively. Under these conditions, the catalyst achieved a propylene conversion of 1.2% and a PO selectivity of 83.7%. It was suggested that the addition of moderate Cu and Cl altered the electronic environment on the Ag surface, facilitating the generation of epoxidation‐active oxygen species and promoting PO formation.

**FIGURE 7:**
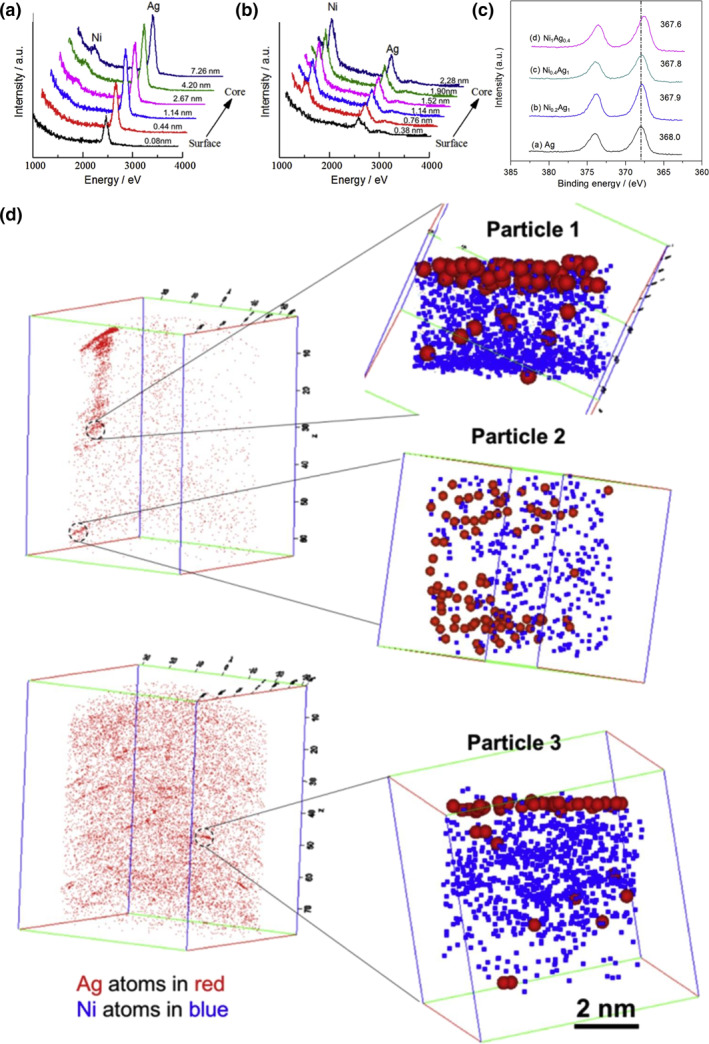
HS‐LEIS spectra of (a) Ni_0.4_Ag_1_ and (b) Ni_1_Ag_0.4_, (c) X‐ray Photoelectron Spectroscopy (XPS) spectra of Ag/SBA‐15 and selected NiAg/SBA‐15 catalysts, (d) Atom Probe Tomography data taken from Ni_1_Ag_0.4_/SBA‐15. Reproduced with permission from Ref. [52]. Copyright 2018, Elsevier.

Nanocatalysts of core‐shell structure have also been successfully applied in the fields such as electrocatalysis,[^44^, ^45^, ^46^, ^47^] selective hydrogenation^[48]^ and oxidation,^[49]^ showing activity several times higher than that of single metal and alloy materials, while maintaining high selectivity. For the chemical selection of hydrogenation of dimethyl oxalate to methyl ethanoate (MG) using a co‐impregnation method,[^50^, ^51^] mesoporous SBA‐15‐supported bimetallic silver‐nickel catalysts (Ag‐Ni/SBA‐15) were prepared, and the yield of MG using the Ag‐Ni/SBA‐15 catalyst exhibited a tenfold increase to 90.6% compared to single‐metal Ag or Ni at 200°C. In a related study, Yu et al.^[52]^ created catalysts with a nickel‐core and silver shell supported by SBA‐15 for the propene epoxidation to PO using oxygen. Utilizing a high‐energy Ne^+^ beam to remove surface atoms to a known depth, the HS‐LEIS depth profiles of Ni_0.4_Ag_1_ and Ni_1_Ag_0.4_ are displayed in Figure 7a,b. These profiles confirm the core‐shell configuration, with Ag peaks appearing before signals from the inner Ni atoms. As depicted in Figure 7c, the 3d5/2 peak exhibited a gradual shift in binding energy from 368.0 to 367.6 eV in Ni_1_Ag_0.4_/SBA‐15 compared to Ag/SBA‐15. This shift to a lower binding energy may be indicative of electron transfer from the Ni phase to the Ag phase. The introduction of nickel increased the distance between the Ag‐Ag bonds, exerting a ligand effect and lattice expansion effect. Figure 7d also illustrates the core‐shell configuration of Ni_1_Ag_0.4_/SBA‐15 with a shell thickness of 1‐3 atomic layers, corresponding to an average Ag thickness of 0.63 nm as determined by HS‐LEIS analysis.

### 2.2 Cu‐based catalysts

For Cu‐based catalysts, determining the active center for propene epoxidation has been a subject of debate. In earlier studies, Li et al.[^53^, ^54^] proposed that Cu^0^ might serve as the active center for propene epoxidation. Similarly, Vaughan et al.^[55]^ conducted propene epoxidation over SiO_2_‐loaded Cu‐based catalysts, achieving high PO selectivity without the need for H_2_ and NaCl modification. They investigated the oxidation state of copper using XPS and Auger spectroscopy, ultimately concluding that Cu^0^ was the active center. In contrast, Wang et al.[^56^, ^57^] identified Cu^+^ as the active center in the direct propene epoxidation by oxygen. Therefore, extensive studies have been conducted on single‐metal Cu‐based supported catalysts, multi‐metallic Cu‐based supported catalysts, and the related crystal plane effects.

Moreover, the coexistence of distinct adsorbed oxygen species (O_2_^−^, O^−^, and O^2‐^) resulting from molecular oxygen activation facilitates the parallel progression of the epoxidation reaction, harnessing either lattice or adsorbed oxygen. DFT calculations have been instrumental in elucidating the underlying mechanism of propylene epoxidation. For this reaction, theoretical investigations are pivotal in revealing reactive oxygen sites and simulating surface oxametallacycle (OMC) intermediates. Notably, Cu exhibits a propensity for oxygen stabilization, fostering the formation of oxide structures under reactive conditions. Consequently, theoretical endeavors on Cu have predominantly focused on unraveling the mechanism and oxidation states pertinent to copper oxide surfaces, as opposed to metallic copper.

Song et al.^[58]^ delved into the CuO surface, contrasting the propene epoxidation behavior on the (111) and (100) facets. Their findings revealed comparable adsorption energies for two distinct propylene OMC intermediates across both facets. On the CuO (111) facet, acrolein formation emerged as more exothermic and kinetically advantageous. In contrast, the (100) facet showcased pathways leading to the generation of PO, acetone, and propane. Moreover, Song et al.^[59]^ further examined the kinetic dynamics on the Cu_2_O (111) surface in an oxygen‐rich environment. Their analysis underscored the strong influence of oxygen species on the activation barriers for OMC formation and ring closure: the weakly basic adsorbed O_2_^−^ molecule selectively promoted PO formation, whereas the highly reactive and basic adsorbed O^−^ atoms favored acrolein generation. Conversely, lattice oxygen O^2‐^ posed a substantial energy barrier for OMC ring closure, indicating its unfavourability for PO production.

The DFT research has significantly enhanced our comprehension of how surface architecture and oxygen species dictate selectivity, furnishing valuable insights for designing oxidation states featuring stable copper and surface oxygen species aimed at enhancing PO selectivity.

#### 2.2.1 Single‐metal Cu‐based supported catalysts

Single metal nanoparticles, as important catalytic activity centers, may undergo agglomeration and deactivation during the preparation process and catalytic reactions, affecting catalytic activity and selectivity. In order to improve the catalytic performance of metal nanoparticles, researchers are committed to introduce some alkali metal ions and transition metal ions into metal nanocatalysts. Through the introduction of other components, it helps to improve the stability and dispersion of metal nanoparticles due to the presence of electronic, anchoring effect and so on. Meanwhile, the pretreatment with different atmosphere and temperature can affect the catalyst particle size. Wang^[60]^ synthesized VO_*x*_‐modified Cu catalysts using a co‐precipitation method. The combination of VO_*x*_ and Cu resulted in a 16% PO selectivity at 2.7% propylene conversion. In this catalyst system, the formation of PO does not require support or conventional additives such as alkali metals and halides. While Cu alone exhibited very low PO generation activity, vanadium oxide alone could not catalyze PO formation. Although the catalyst's performance still falls short, the study of the vanadium modification provides valuable insights into the active site of copper for propene epoxidation. To gain a deeper understanding of the active site of VO_*x*_‐modified Cu catalysts, they investigated the effect of pretreatment on catalytic performance. Characterization results revealed that vanadium modification enhanced copper dispersion and promoted the transformation of metal Cu to Cu_2_O, thereby enhancing catalytic activity. Combining these characterizations with catalytic results, they concluded that Cu_2_O serves as the active site for propene epoxidation.

Subsequently, they found that the Cs^+^‐5wt%CuO_*x*_/SiO_2_ catalyst exhibited the highest PO selectivity and PO yield, reaching 34% and 2.6%, respectively, among a series of alkali metal ion‐modified CuO_*x*_/SiO_2_ catalysts, while maintaining a propylene conversion of 7.5%.^[61]^ Characterization results indicated that alkali metal ion modification reduced the size of CuO_*x*_ nanoparticles. Cs^+^ modification inhibited the ongoing PO conversion by reducing the catalyst's acidity and promoted PO production by decreasing lattice oxygen activity. These observations pointed to Cu^+^ generated during the reaction as the active center for propene epoxidation with oxygen.

A novel catalyst based on copper supported on titania (Cu‐OH‐Cl‐TiO_2_) was prepared using a slurry impregnation method.^[62]^ This catalyst displayed activity and selectivity for propene epoxidation, achieving a propylene conversion of 4.8% and a PO selectivity of 38.9% at 500K. Characterization results revealed that Cu^2+^ rather than Cu^+^ cation served as the active site for propylene conversion in the Cu‐OH‐Cl‐TiO_2_ catalyst. The variation in the amount of chloride ions had a negligible effect on catalytic activity. Spectroscopic and X‐ray diffraction characterization showed that during the catalytic reaction, Cu^2+^ was converted to CuCl, which could be reverted to Cu_2_(OH)_3_Cl through water and oxygen treatment, allowing the catalyst to be regenerated after the reaction.

Unusually, Su et al.^[63]^ investigated the effect of particle size on unloaded CuO_*x*_ catalysts and found that the highest catalyst activity was achieved when the particle size was 41 nm. Notably, the trend of descending PO selectivity over CuO_*x*_ catalysts with N_2_ pretreatment was significantly slower compared to H_2_ pretreatment or no pretreatment. The CuO_*x*_ catalysts with N_2_ pretreatment exhibited the highest epoxidation activity. Additionally, the XRD patterns of CuO_*x*_ catalysts pretreated in N_2_ at different temperatures demonstrated that the relative content of Cu_2_O and Cu^0^ species in CuO_*x*_ catalysts remained largely unchanged over the entire range of N_2_ pretreatment temperatures. TEM images of CuO_*x*_ catalysts with varying N_2_ pretreatment temperatures revealed changes in the morphology and size of copper species, with larger particles formed at higher N_2_ pretreatment temperatures. Hence, they concluded that the significant enhancement in PO selectivity was primarily due to the size difference of Cu species rather than the valence state of Cu.

#### 2.2.2 Multi‐metallic Cu‐based supported catalysts

Bimetallic catalysts, which often exhibit significantly different electronic and chemical properties from the parent metals, offer an opportunity to obtain novel catalysts with enhanced selectivity, activity and stability. Bimetallic catalysts are widely used in many catalytic processes. In the field of propene epoxidation, Seubsai's group has conducted extensive research on multi‐metallic catalysts.[^64^, ^65^, ^66^, ^67^, ^68^, ^69^, ^70^, ^71^] In the beginning, Kahn et al.^[64]^ employed newly developed array channel microreactors for the rapid screening of numerous catalytic materials. The initial screening experiments identified Cr, Mn, Cu, Ru, Pd, Ag, Sn, and Ir supported on silica as particularly promising for PO generation. Subsequent experiments revealed that the bimetallic Cu‐on‐Mn/SiO_2_ catalyst significantly increased PO yield compared to single‐metal catalysts. Although bimetallic catalysts are commonly used for propene epoxidation, these systems exhibit low propylene conversion.

**FIGURE 8:**
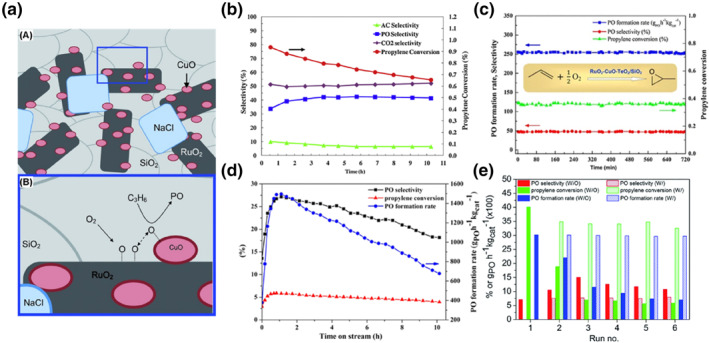
(a) (A) Schematic structure of the RuO_2_‐CuO‐NaCl/SiO_2_ catalyst, (B) Proposed mechanism for the epoxidation of propylene, (b) Time‐on‐stream testing results Sb_2_O_3_‐CuO‐NaCl/SiO_2_ catalysts, (c) Time‐on‐stream testing results RuO_2_‐CuO‐TeO_2_/SiO_2_ catalysts, (d) Time‐on‐stream testing of the optimal RuO_2_‐CuO‐NaCl‐TeO_2_‐MnO_*x*_/SiO_2_ catalysts, (e) Multiple test runs of the optimal RuO_2_‐CuO‐Cs_2_O‐TiO_2_/SiO_2_ catalyst with (w/) and without (w/o) treating with fumed HCl under the optimal operating condition for Propylene oxide (PO) formation rate. Reproduced with permission from Ref. [73]. Copyright 2014, Wiley. Reproduced with permission from Ref. [66]. Copyright 2015, Elsevier. Reproduced with permission from Ref. [68]. Copyright 2018, Springer. Reproduced with permission from Ref.[69]. Copyright 2017, American Chemical Society. Reproduced with permission from Ref.[70]. Copyright 2016, Royal Society of Chemistry.

A combinatorial micro‐reactor is used to investigate various bimetallic catalytic systems and their effects on the direct gas‐phase propene epoxidation to PO using molecular oxygen.^[65]^ Among the bimetallic catalytic systems containing Ag, Ru, Mn, and Cu metals, the Mn‐Cu/c‐SiO_2_ bimetallic system exhibited high PO selectivity and significantly increased the PO yield. The highest propylene conversion and PO selectivity were observed for the 2% Cu/5% Ru/c‐SiO_2_ catalyst. Furthermore, propylene conversion reached 9.6%, and PO selectivity increased fivefold from 7.1% to 36% with a yield of 3.46% after NaCl modification at 300°C. NaCl acted solely as an electronic agent and had no effect on particle size. Similarly, Zhang et al.^[72]^ achieved a 5.2% propylene conversion and 36% PO selectivity at 225°C on the RuO_*x*_‐CuO_*x*_/SiO_2_ catalyst. When only Cu or Ru was loaded on SiO_2_ individually, the main product of the propylene oxidation reaction was acrolein, suggesting a synergistic effect between Cu and Ru in the propene epoxidation reaction. Furthermore, Seubsai et al.[^67^, ^73^] reported a new SiO_2_‐supported trimetallic RuO_2_‐CuO‐NaCl catalyst for the direct propene epoxidation using molecular oxygen, achieving 40%–50% PO selectivity at 10%–20% propylene conversion. Only the trimetallic combination of Ru, Cu, and Na, namely RuO_2_‐CuO‐NaCl/SiO_2_, exhibited the best PO selectivity of 49% and propylene conversion of 14%. Under the same experimental conditions, the single‐metal and binary‐metal combinations exhibited poor epoxidation performance. This suggests that small crystalline CuO particles close to RuO_2_ act as the catalytically active site for PO synthesis. In the reaction mechanism, as shown in Figure 8a, an O_2_ molecule is initially chemisorbed on an active center on the RuO_2_ surface and dissociates into two surface O atoms (Oa). Oa migrates through the surface to the adjacent CuO site to form CuO‐O. Gas‐phase propylene interacts with CuO‐O to form an epoxidation intermediate, ultimately leading to PO formation.

A series of multi‐metallic catalysts,[^66^, ^68^, ^69^, ^70^, ^71^] such as Sb_2_O_3_‐CuO‐NaCl/SiO_2_ catalysts, RuO_2_‐CuO‐TeO_2_/SiO_2_ catalyst, RuO_2_‐CuO‐NaCl‐TeO_2_‐MnO_*x*_/SiO_2_ catalyst, and RuO_2_‐CuO‐Cs_2_O‐TiO_2_/SiO_2_ catalyst, were prepared based on the RuO_2_‐CuO/SiO_2_ catalyst (Figure 8b–e). Among them, the Cs_2_O and TiO_2_‐modified catalyst exhibited a very high PO generation rate of 3015 g kg_cat_^−1^ h^−1^. The RuO_2_‐CuO‐TeO_2_/SiO_2_ catalyst maintained its high activity during a 12‐h stability test without signs of deactivation.

#### 2.2.3 Crystal plane effect

**FIGURE 9:**
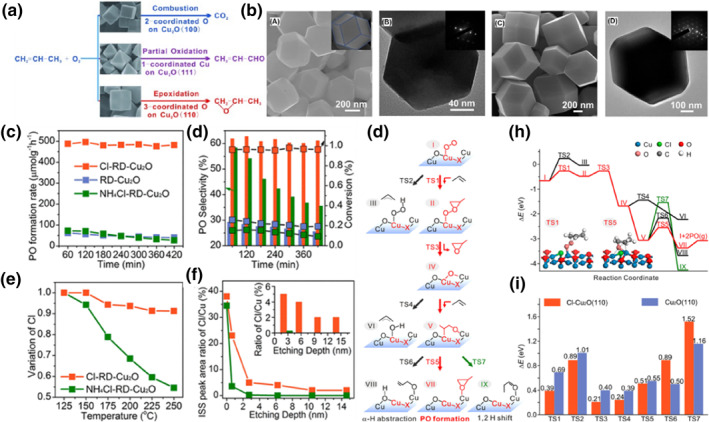
(a) Crystal‐plane‐controlled selectivity of Cu_2_O catalysts in the oxidation of propylene by molecular oxygen. (b) Structure characterization of RD‐Cu_2_O and Cl‐RD‐Cu_2_O nanocrystals. (A) SEM image of Cl‐RD‐Cu_2_O. (B) TEM image of Cl‐RD‐Cu_2_O and corresponding SAED pattern (inset). (C) SEM image of RD‐Cu_2_O. (D) TEM image of RD‐Cu_2_O and corresponding SAED pattern (inset). (c) Stability of RD‐Cu_2_O, Cl‐RD‐Cu_2_O and NH_4_Cl‐RD‐Cu_2_O, (d) Variation of Propylene oxide (PO) selectivity (histogram) and conversion (scatter plot) with time at 200°C, (e) Cl loss of Cl‐RD‐Cu_2_O and NH_4_Cl‐RD‐Cu_2_O during the catalysis process, (f) Area ratio of the Cl/Cu peak with different etching depths of Cl‐RD‐Cu_2_O and NH_4_Cl‐RD‐Cu_2_O from HS‐LEIS, (g) Schematic diagram of the reaction mechanism, and (h) potential energy distribution of the reaction of propylene and O_2_ on the surfaces of Cl‐Cu_2_O (110), (i) Comparison of the energy barriers of direct epoxidation of propylene on the Cl‐Cu_2_O (110) and Cu_2_O (110) surfaces. Reproduced with permission from Ref. [80], Copyright 2014, Wiley. Reproduced with permission from Ref. [81]. Copyright 2020, American Chemical Society.

The crystal structure of the catalyst surface significantly influences its catalytic activity, making the crystal plane effect of metal oxide a topic of great interest. Metal oxide nanoparticle catalysts with exposed high‐activity planes have been designed and synthesized for various applications. For instance, Co_3_O_4_ has been used in electrocatalytic full water splitting,^[74]^ Li‐O_2_ batteries,[^75^, ^76^] CO oxidation,^[77]^ styrene oxidation,^[78]^ and as electrochemical H_2_O_2_ sensors.^[79]^ In 2014, Huang et al.^[80]^ reported that the selectivity of oxide catalysts in complex reactions, such as propylene and oxygen oxidation, could be controlled by changing their morphology. As shown in Figure 9a, they proposed that Cu_2_O octahedra (o‐Cu_2_O) with exposed (111) crystal faces were the most selective for acrolein, Cu_2_O cubes (c‐Cu_2_O) with exposed (100) crystal faces were the most selective for carbon dioxide, and Cu_2_O rhombic dodecahedra (d‐Cu_2_O) with exposed (110) crystal faces were the most selective for PO. The catalytic active sites for the production of acrolein, PO, and carbon dioxide were single coordination Cu^+^ (111) on Cu_2_O, triple coordination O (110) on Cu_2_O, and two coordination O (100) on Cu_2_O, respectively. There is room for improving the conversion of propylene by reducing the size and increasing the specific surface area of d‐Cu_2_O nanocrystalline catalysts, making the Cu_2_O rhombic dodecahedra a promising catalyst for the selective generation of PO and acrolein.

A few years later, Huang et al.^[82]^ investigated the propene epoxidation on cubic Cu_2_O nanocrystals (c‐Cu_2_O NCs) of different sizes. They found that the sizes of various c‐Cu_2_O NCs had a significant effect on the catalytic performance of propylene oxidation with oxygen. They successfully identified 27 nm‐sized cubic Cu_2_O nanocrystals with (100) facets and (110) edges as highly selective catalysts for propene epoxidation, achieving over 80% PO selectivity at low temperatures (90–110°C).

Wang et al.^[83]^ synthesized Cu_2_O nanocubes (Cu_2_O‐NCs) enclosed by (100) facets using a surfactant‐free wet chemical method and explored the effect of Cl^−^ on the direct propene epoxidation by molecular oxygen on Cu_2_O‐NCs. The results showed a volcano‐type relationship between Cl^−^ loading and PO selectivity, with 0.33 wt% NH_4_Cl‐Cu_2_O‐NCs exhibiting the highest PO selectivity, reaching 57.2% and 48.7% at 125°C and 150°C, respectively. They also observed a similar relationship between propylene conversion and NH_4_Cl, with the highest conversion achieved at 0.03 wt% loading. Combining selectivity and conversion, the 0.33 wt% NH_4_Cl‐Cu_2_O‐NCs outperformed all Cu_2_O catalysts, with a TOF value of 3.4 × 10 s^−1^ for PO at 150°C. TPD‐MS experiments were used to determine the interfacial relationship between NH_4_Cl and Cu_2_O, indicating that part of Cl^−^ might be embedded in the Cu_2_O lattice. Additionally, the loss of Cl^−^ from the catalyst surface was found to cause severe degradation in the catalytic performance of olefin epoxidation.

Wang et al.^[81]^ discovered that when NH_2_OH‐HCl was used as the reducing agent, a small amount of Cl would remain in the Cl‐RD‐Cu_2_O crystal growth process. CuCl and Cu_2_O have similar structural features, with Cu atoms arranged at the face center cubic sites and anions tetrahedrally coordinated to the Cu atoms. It has been shown that part of the lattice oxygen atoms in Cu_2_O can be replaced by Cl atoms. This method is referred to as the “intergrowth method.” As shown in Figure 9b, SEM and TEM images revealed that both Cl‐RD‐Cu_2_O and RD‐Cu_2_O have a dodecahedral crystal shape. In addition, SAED confirmed exposure of (100) facets. Although having a similar morphology and structure, the PO formation rate of Cl‐rhombic dodecahedral Cu_2_O (Cl‐RD‐Cu_2_O) is one order of magnitude higher than that of Cl‐free RD‐Cu_2_O and ammonium chloride solvent post‐treatment (NH_4_Cl‐RD‐Cu_2_O). Cl‐RD‐Cu_2_O exhibited a PO selectivity of 63% at 200°C with a TOF of 12.0 h^−1^ and demonstrated excellent stability (Figure 9c). In the catalytic performance comparison, Cl‐OCT‐Cu_2_O > NH_4_Cl‐OCT‐Cu_2_O > OCT‐Cu_2_O was observed. Octahedral Cu_2_O (OCT‐Cu_2_O) and Cl‐OCT‐Cu_2_O were prepared using the intergrowth method, and NH_4_Cl‐OCT‐Cu_2_O was prepared using the post‐treatment method. The trend was consistent with the other catalysts: Cl‐OCT‐Cu_2_O > NH_4_Cl‐OCT‐Cu_2_O > OCT‐Cu_2_O.

In Figure 9e–f, further insights into the differences in the nature of Cl in NH_4_Cl‐RD‐Cu_2_O and Cl‐RD‐Cu_2_O were illustrated using quasi in‐situ XPS and HS‐LEISS. Quasi in‐situ XPS revealed that over 45% of Cl in NH_4_Cl‐RD‐Cu_2_O is lost as the temperature increases to 250°C, while the loss of Cl in Cl‐RD‐Cu_2_O is negligible. Additionally, Cl was observed to penetrate the Cu_2_O lattice to a depth of tens of nanometers. In Figure 9g–i, DFT calculations provided evidence that the substituted Cl promoted the formation of electrophilic oxygen and enhanced the production of PO, underscoring the significance of regulating the active site through anionic doping. These findings demonstrate that doping Cl into the lattice of Cu_2_O nanocrystals through the intergrowth method not only enhances the catalytic selectivity and conversion of the direct propene epoxidation reaction but also resolves the long‐standing issue of Cl loss. This work offers a strategy for developing catalysts and exploring additive effects by doping well‐defined nanocrystals with uniformly separated anions to activate nearby.

### 2.3 Catalysts containing Mo, Bi, Ti, W, and Fe

In addition to the more common silver‐based and copper‐based metal catalysts, other metals such as Mo, Bi, and Ti have also been explored for propene epoxidation. A catalyst containing Ti oxide dimers on silica was found for radical production which promotes selective epoxidation of propylene.^[84]^ A possible reaction mechanism for the efficient generation of radicals by stabilizing the Ti^3+^ state is proposed.

It is also found that MoO_*x*_ loaded on silica exhibited high activity for propene epoxidation.^[85]^ They suggested that the catalytic post‐bed volume played a crucial role in PO formation. On the MoO_*x*_/SiO_2_ catalyst, propylene conversion reached 17.6%, with a PO selectivity of 43.6% under conditions of 5 atm, 300°C, and C_3_H_6_/O_2_/He = 10/5/10 cm^3^ min^−1^. Their conclusion was that the active species in the MoO_*x*_/SiO_2_ sample was a crystalline molybdenum trioxide species effective in extracting hydrogen atoms from propylene to generate radicals which subsequently react with molecular oxygen, but inefficient at inserting lattice oxygen. The formed radicals could desorb into the gas phase and subsequently react with molecular oxygen to form PO. Wei et al.^[86]^ discovered that modifying the double mesoporous material Bi_2_SiO_5_/SiO_2_ with molybdenum oxide significantly enhanced its activity and selectivity for PO generation in 2014. Under relatively mild conditions with a Mo/Bi ratio of 5, a favorable synergistic effect between Mo and Bi was achieved, resulting in a PO selectivity of 55.14% at 21.99% propylene conversion.

**FIGURE 10:**
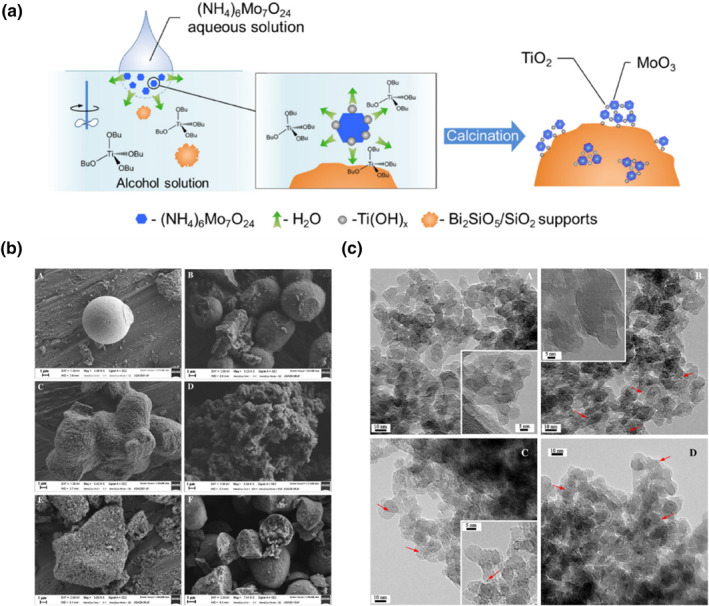
(a) Illustration of synthesis mechanism of highly dispersed amorphous MoO_3_. (b) FE‐SEM of the fresh samples. Samples (A) SiO_2_, (B) Fe_0.005_SiO_2_, (C) Fe_0.01_SiO_2_, (D) Fe_0.02_SiO_2_, (E) Fe_0.03_SiO_2_ and (F) Fe_impreg_SiO_2_. (c) TEM images of the fresh catalysts. (A) Fe_0.005_SiO_2_, (B) Fe_0.01_SiO_2_, (C) Fe_0.03_SiO_2_ and (D) Fe_impreg_SiO_2_. Reproduced with permission from Ref. [88], Copyright 2016, Wiley. Reproduced with permission from Ref. [89]. Copyright 2016, Elsevier.

A subsequent catalyst was reported with Ti modification on MoO_3_‐Bi_2_SiO_5_,[^87^, ^88^] which catalyzed propene epoxidation more efficiently with O_2_ at a lower Mo loading. On this catalyst, a Mo/Bi (Ti/Mo = 0.3) ratio of 3 yielded a 20.5% propylene conversion, 64.7% PO selectivity, and a 6.6 mmol gcat^−1^ h^−1^ PO generation rate at 0.15 MPa and 400°C. They proposed a one‐step synthesis strategy for highly dispersed amorphous MoO_3_ over support catalysts, as depicted in Figure 10a. The addition of Ti constrained MoO_3_ within the “Ti(OH)_*x*_” cage, inhibiting the formation of Bi_2_Mo_3_O_12_, and enhancing the formation of highly dispersed molybdenum trioxide active sites. This was beneficial for improving the PO selectivity and propylene conversion of the Ti‐modified catalyst. Since the reaction requires high temperatures leading to significant energy consumption and the Ti‐modified catalyst is sensitive to temperature, a more efficient promoter is needed to reduce energy consumption.

Wang et al.[^90^, ^91^] discovered that the addition of phosphorus (P) could modulate the hydrogenation and deoxygenation activity of amorphous catalysts, enabling the catalysts to maintain high p‐cresol hydrodeoxygenation activity at lower temperatures. Therefore, Chen et al.^[92]^ attempted to introduce P into the MoO_3_‐Bi_2_SiO_5_/SiO_2_ catalyst, which significantly improved the performance of propene epoxidation. The modification of the MoO_3_‐Bi_2_SiO_5_/SiO_2_ catalyst with P also acted as a promoter, leading to the highly dispersed nature of MoO_3_ and a reduced reaction temperature of 360°C. Additionally, the P modification clearly increased the number of weak and moderate acid sites, which was conducive to improving the selectivity for propylene oxide.

WO_*x*_/ZrO_2_ catalysts are known for their excellent thermal stability and remarkable redox performance,[^93^, ^94^] while CeO_2_ has oxygen storage/release capacity,^[95]^ both of which are of interest in the field of catalysis. Lee et al.^[96]^ prepared tungsten oxide loaded on Ce_0.05_Zr_0.95_O_2_ (CZ) support by precipitation, followed by wet impregnation to create WO_*x*_/CeO_2_(C), WO_*x*_/CZ, and WO_*x*_/ZrO_2_(Z) catalysts for the propene epoxidation reaction with oxygen. In the catalytic performance test for propene epoxidation, it was observed that pure CeO_2_ as a support favored the conversion of propylene, while pure ZrO_2_ as a support exhibited high selectivity for PO. Notably, the acidity of the WO_*x*_/CZ catalyst falls between that of WO_*x*_/C and WO_*x*_/Z. The authors proposed that the good performance of the WO_*x*_/CZ catalyst, with 3.8% propylene conversion and 46.8% PO selectivity, is attributed to its moderate reduction ability and optimal acid‐base ratio. It can be speculated that the acidic site affects the electronic property of oxygen on WO_3_, which is involved in formation of propylene oxide. The basic site reacts with allylic hydrogen of propylene to form CO_2_ and H_2_O.

García‐Aguilar et al.^[89]^ proposed a simple sol‐gel method for the one‐step synthesis of well‐dispersed Fe_0.0*x*_SiO_2_ catalysts, which demonstrated high activity and PO selectivity in the propene epoxidation reaction. As shown in Figure 10b, the catalyst is an unformed continuous material with high surface roughness. Figure 10c reveals some particles with a size of around 1 nm, indicated by the red arrows. The iron species in the catalyst were distinguished using FTIR, UV‐Raman, and UV‐VIS characterization techniques. They found that iron was dispersed on silicon frames with surface iron atoms exhibiting tetrahedral or pseudo‐tetrahedral coordination and small iron oxide particles on the silicon surface, particularly in samples with high iron load. According to their evidence, it was proposed that the oxygen absorbed on the well‐dispersed iron species incorporated in the silica framework and the propylene absorbed on the acidic protons to give the desired epoxide. While iron oxide particles can hinder PO generation, they proposed a strategy to initially eliminate these iron oxide particles, involving a straightforward post‐treatment of the catalyst with alkali or alkaline earth elements (such as K or Ca) to enhance the selectivity of the iron‐based catalyst for PO.^[97]^ Experiments confirmed that the addition of K and Ca altered the physicochemical properties of the catalyst, reduced its surface acidity, and prevented carbon deposition on the catalyst. K was found to be more efficient than Ca in removing iron oxide particles. Consequently, the K‐modified Fe‐SiO_2_ catalyst achieved a 65.5% PO selectivity without producing excessive organic by‐products.

## 3 SINGLE‐ATOM CATALYSTS

Single‐atom catalysts (SACs) have garnered significant attention owing to their high atom utilization efficiency, stable active sites, and outstanding catalytic performance when compared to conventionally supported nanoparticles. Moreover, the robust interaction between isolated metal atoms and supports facilitates excellent stability, and the high dispersion of metal sites in SACs aids in the precise identification and characterization of active centers. SACs hold a pivotal role in numerous fields, including applications in single‐atom catalyzed hydroformylation and epoxidation reactions.[^98^, ^99^, ^100^, ^101^, ^102^] Recently, Yang et al.^[103]^ summarized the recent advances in the adsorption, activation and reaction exploiting novel catalytic materials (single atoms, nanoclusters and nanoparticles) in four challenging selective oxidation reactions, including selective oxidation of methane, aerobic oxidation of alcohols, epoxidation of alkenes, and preferential oxidation of carbon monoxide in hydrogen. The electronic and geometrical structures of single atoms, nanoclusters and nanoparticles are discussed and an in‐depth understanding of the active species, active structures and conformational relationships in these catalytic systems is presented.

### 3.1 Mo SACs

Mo atoms can coordinate with other metal atoms to form Mo‐O‐Metal structures, which could effectively catalyze propene epoxidation using molecular O_2_. Chen et al.^[104]^ incorporated transition metal Cu into the MoO_3_‐Bi_2_SiO_5_/SiO_2_ catalyst through a simple co‐impregnation process, resulting in a significant enhancement of propene epoxidation performance. The rate of PO generation increased from 106 to 336 g kg_cat_^−1^ h^−1^. Characterization results revealed that the addition of Cu effectively boosted the catalytic activity of propylene at low temperatures, primarily attributed to the formation of a metastable Mo‐O‐Cu solid solution state with oxygen vacancies at the active sites.

In addition to introducing additives to MoO_3_‐Bi_2_SiO_5_/SiO_2_, Chen et al.^[105]^ proposed an effective strategy to achieve Mo‐O‐Bi coordination by strongly anchoring molybdenum trioxide on the surface. This approach improved its dispersion without requiring the addition of other additives and ensured good stability. The unique feature of this catalyst is the pretreatment of Bi_*x*_Si_1−*x*_O_2_ with acetic acid solution before the preparation of MoO_3_/Bi_x_Si_1−*x*_O_2_. This process offers the advantage of eliminating surface BiO_*x*_ clusters, exposing highly dispersed framework Bi ions and defects, which facilitates Mo fixation. Acid pretreatment can remove the self‐passivation layer and expose more acidic sites. Ultimately, this catalyst achieved the best performance: 20.4% propylene conversion and 73.2% PO selectivity.

### 3.2 Cu SACs

Mixed oxides, such as spinel or perovskites, offer stability, highly ordered structures, and unique electronic and physical/chemical properties. Perovskite oxides with the general formula ABO_3_ have garnered significant attention in catalytic reactions due to their structural stability and adaptable electron mobility. In the perovskite structure, A represents a rare‐earth or alkaline cation, characterized by 12‐fold coordination with oxygen anions, while B is a 3d transition metal with 6‐fold coordination with oxygen anions.[^106^, ^107^, ^108^, ^109^, ^110^, ^111^] The substitution of B sites can be used to control the valence electronic structure of bulk oxides, optimize the binding strength of surface reactants or intermediates, and even adjust the primary reactive oxygen species. Notably, mixed oxides derived from TiCuO_*x*_ have demonstrated high catalytic activity in CO oxidation, with copper being stabilized as Cu^+^.[^112^, ^113^] The stability of TiO_*x*_ to Cu^+^ suggests that TiCuO_*x*_ holds promise for selective propene epoxidation. Thus, Yang et al.^[114]^ conducted a comparative study of propene epoxidation activity on four surfaces: Cu (111), Cu_2_O, and TiCuO_*x*_ containing 0.6 ML (monolayer) and 0.9 ML TiO_*x*_ in 2015. The TiCuO_*x*_ surface exhibited higher activity than Cu (111) or Cu_2_O, with the highest activity observed at 0.6 ML TiO_*x*_ surfaces, resulting in a PO selectivity of 69%, approximately 30% higher than the PO selectivity exhibited by the other three surfaces. This highlights the exceptional activity and selectivity of the TiCuO_*x*_ surface for propene epoxidation. The modification of Cu_2_O with TiO_2_ appears to be an effective approach to stabilize Cu^+^ and reduce oxide basicity, making TiCuO_*x*_ a promising catalyst for propene epoxidation.

Lanthanum‐based perovskite oxides have been extensively researched in various catalytic systems, demonstrating outstanding performance in NO oxidation,^[108]^ soot combustion,^[111]^ ammonia borane dehydrogenation,^[115]^ and steam reforming of biomass tar for hydrogen production.^[116]^ In 2021, Li^[117]^ prepared LaCo_*x*_Cu_1−*x*_O_3−*δ*_‐NaCl catalysts using the citric acid sol‐gel method and xCuO/LaCoO_3_‐NaCl catalysts using the conventional deposition method as comparison samples. These perovskite materials were successfully employed for the direct propene epoxidation with oxygen.

**FIGURE 11:**
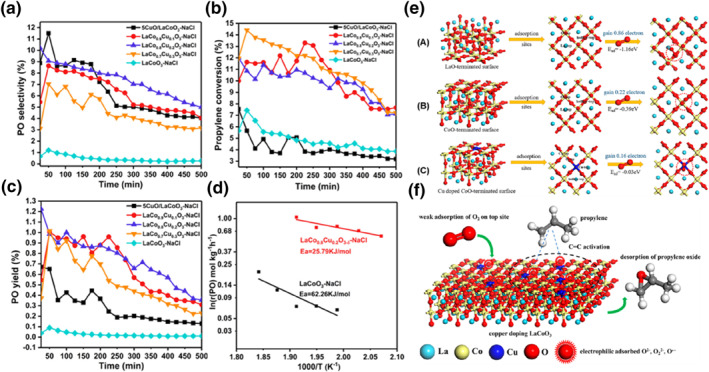
(a) Propylene oxide (PO) selectivity, (b) propylene conversion, and (c) PO yield with time for different catalysts at 250°C. (d) Apparent activation energy of LaCoO_3_‐NaCl catalysts doped and undoped with Cu. (e) Stimulated oxygen adsorption energy on various adsorption: (A) LaO terminal surface, (B) CoO terminal surface, (C) CuO terminal surface. (f) Reaction mechanism of direct epoxidation of propylene by molecular oxygen over LaCo_*x*_Cu_1−*x*_O_3−*δ*_ catalyst. Reproduced with permission from Ref. [117]. Copyright 2021, American Chemical Society.

**FIGURE 12:**
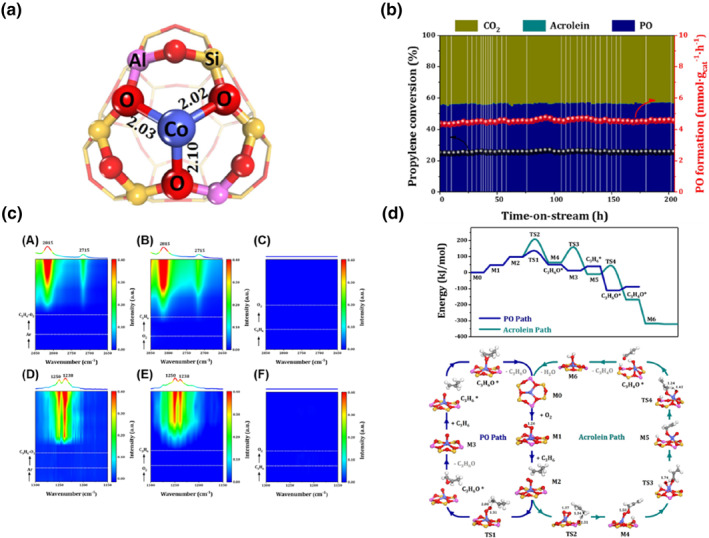
(a) Structure of Co@Y, (b) Stability test of Co@Y catalyst in propene epoxidation at 500°C, (c) (A) In situ FT‐IR spectra of Co@Y recorded in flowing C_3_H_6_‐O_2_ at 573K; (B) In situ FT‐IR spectra of Co@Y after the successive feeding of O_2_ and C_3_H_6_ at 573K; (C) In situ FT‐IR spectra of Co@Y after the successive feeding of C_3_H_6_ and O_2_ at 573K; (D) In situ FT‐IR spectra of Co@Y recorded in flowing C_3_H_6_‐O_2_ at 673K; (E) In situ FT‐IR spectra of Co@Y after the successive feeding of O_2_ and C_3_H_6_ at 673K; (F) In situ FT‐IR spectra of Co@Y after the successive feeding of C_3_H_6_ and O_2_ at 673K, (d) Reaction mechanism of propene epoxidation with molecular oxygen over Co@Y. Reproduced with permission from Ref. [9]. Copyright 2022, American Chemical Society.

As depicted in Figure 11a–d, the pure LaCoO_3_ sample exhibited a PO selectivity below 2%. However, the crystalline LaCo_*x*_Cu_1−*x*_O_3−*δ*_ (0.1 ≤ *x* ≤ 0.9) samples demonstrated significantly increased PO selectivity. Additionally, the loading of Cu_2_O on LaCoO3 also yielded high PO selectivity. Among the catalysts, the LaCo_0.8_Cu_0.2_O_3−*δ*_ catalyst achieved the highest PO yield at 1.3% and a PO generation rate of 60.4 g kgcat^−1^ h^−1^. This catalyst enabled a PO selectivity of 10.8% and a propylene conversion of 12.0% even within a 500‐min timespan. Figure 12d illustrates that effective Cu doping reduced the apparent activation energy of the PO generation rate from 62.26 kJ/mol to 25.79 kJ/mol, enhancing the catalytic performance of the catalyst for direct epoxidation at lower temperatures. Based on these experimental results, it is suggested that the propene epoxidation reaction over Cu‐containing perovskite catalysts may benefit from the coexistence of different valence states of Cu.

As depicted in Figure 11e, the calculation of oxygen adsorption on three types of surfaces was conducted. It is evident that the LaO terminal surface exhibits a higher oxygen adsorption capacity compared to the CoO terminal surface, which is not conducive to the dissociation of oxygen for attacking the C=C double bond. Consequently, the propene epoxidation reaction predominantly takes place at the CoO terminal surface. Finally, as illustrated in Figure 11f, a reaction mechanism is proposed based on the amalgamation of characterization and DFT calculations. When Cu is anchored to the CoO terminal surface, the O_2_ adsorption site shifts from Co‐terminal to Cu‐terminal, leading to weakened oxygen adsorption, which promotes oxygen dissociation. Furthermore, partial substitution of Co with Cu enhances the electrophilicity of oxygen, making it more favorable for activating the C=C bond in propylene. The activated propylene subsequently reacts with the O_2_ physically adsorbed on top of the perovskite to form PO or acetone. Moreover, the weak O_2_ adsorption facilitates the desorption of PO in contrast to pure LaCoO_3_, preventing further oxidation of PO. Copper‐doped LaCoO_3_ facilitates charge transfer and establishes strong interactions between Co and Cu by sharing oxygen atoms, indicating the possible presence of Cu‐O‐Co species and oxygen vacancies on the LaCo_0.8_Cu_0.2_O_3−*δ*_ surface. The anchoring of Cu contributes to the reduction of the number of electrons gained by oxygen and augments its electrophilicity. The LaCo_0.8_Cu_0.2_O_3−*δ*_ sample modified with NaCl features a more abundant presence of moderately basic sites, which enhances the electrophilicity of the catalyst.

Subsequently, they prepared a series of manganese‐doped La_2_CuO_4_ perovskite (LaMn_*x*_Cu_1−*x*_O_3_) catalysts with adjustable electronic structures, and LaMn_0.5_Cu_0.5_O_3_ displayed the best performance at 150°C with a PO selectivity of 74.2% and a PO generation rate of 0.239 mol·kgcat^−1^ h^−1^, albeit with propylene conversion below 0.02%.^[118]^ The results indicate that the doping of Mn (strong binding energy) into the La_2_CuO_4_ (weak binding energy) perovskite catalyst significantly improves its activity and selectivity for the direct epoxidation of propylene. Mn doping on the B‐site of the perovskite increases electron density on the Cu‐site via electron transfer from Mn ions to Cu ions, thus reducing the oxygen adsorption energy on the catalyst surface. Furthermore, Mn doping raises the binding energy of the Cu‐O bond in La_2_CuO_4_. A potential Mn modification mechanism is postulated: the Cu‐O‐Mn site may act as the active site, with Mn ions regulating the electronic properties of the Cu site without being directly involved in the reaction. As the electronic structure evolves, the oxygen activation site transitions from an oxygen vacancy to Cu^+^, favoring the generation of electrophilic oxygen species. The synergistic interaction between Mn and Cu enhances propene epoxidation. Mn doping lowers the apparent activation energy from 82.6 kJ/mol to 39.5 kJ/mol, indicating that Mn substitution also improves the low‐temperature reactivity of the catalyst for the direct propene epoxidation. Both studies on the metal‐doped perovskite support Cu^+^ as the active site for epoxidation.

### 3.3 Co SACs

Zeolites find wide applications as detergents, adsorbents, and catalysts in various petrochemical processes such as cracking, isomerization, alkylation, and epoxidation, harnessing the catalytic power of their Brønsted or Lewis acid sites. Heteroatoms like Fe, Co, and Ti can be incorporated into zeolites, enhancing the charge modulation of the zeolite framework and effectively tailoring its catalytic performance.[^119^, ^120^, ^121^, ^122^]

Li et al.^[9]^ engineered an octahedral zeolite featuring a solitary cobalt ion (Figure 12a), referred to as Co@Y, for the catalytic epoxidation of propylene through an in‐situ hydrothermal approach. Co@Y exhibited unprecedented performance in propene epoxidation, achieving a groundbreaking feat of propene epoxidation using oxygen. Co@Y demonstrated a propylene conversion of 24.6%, a PO selectivity of 57%, and a PO generation rate of 4.7 mmol gcat^−1^ h^−1^ at 500°C. Remarkably, Co@Y exhibited remarkable catalytic stability, retaining its activity over 200 h (Figure 12b). However, at a relatively low temperature of 400°C, there was a gradual decline in activity within 200 h, possibly attributed to the deposition of coke resulting from the condensation of the byproduct acrolein. Furthermore, Li also synthesized Co‐Y and Co/Y using conventional ion exchange and impregnation methods, respectively. Under identical reaction conditions, the Co/Y and Co‐Y catalysts displayed lower PO selectivity and PO formation rates, with PO formation rates falling below 0.5 nmol gcat^−1^ h^−1^.

Based on the in situ FT‐IR spectra of the propene epoxidation reaction in Figure 12c, the formation of PO is contingent on the sequence in which propylene and O_2_ are introduced. Initially, oxygen adsorbs onto the coordination‐unsaturated cobalt site of Co@Y, followed by desorption at temperatures ranging from 500 to 600 K. This process precludes the reaction between weakly adsorbed O_2_ and propylene for PO production, while chemisorbed O_2_ reacts with propylene to yield PO at 773 K. Changes in Co sites during the reaction were elucidated through XANES analyses. The aerobic epoxidation of propylene, with the inclusion of O_2_ and propylene, establishes a redox process involving a Co^2+^‐Co^δ+^‐Co^2+^ (2 < *δ* < 3) cycle, as deduced from FT‐IR analyses.

As depicted in Figure 12d, the reaction mechanism for propene epoxidation with molecular oxygen on Co@Y unfolds as follows: O_2_ and propylene adsorb onto the catalyst, commencing from the active site structure of the Lewis acidic zeolite. Here, the terminal methyl group of propylene initially engages with an oxygen atom in O_2_ to generate a PO molecule, while another oxygen atom is highly reactive towards propylene, directly yielding PO between propylene and oxygen atom. In an alternate scenario, the framework oxygen can act as a Brønsted base site, activating the terminal methyl group of propylene to form an allyl. The allyl then directly combines with an oxygen molecule, resulting in the C_3_H_5_OO species at the cobalt site. The cleavage of the O‐O bond is exothermic with an enthalpy barrier of 82 kJ/mol. Subsequent to the formation of C_3_H_5_O at the cobalt site, the active single oxygen atom can react with a hydrogen atom in C_3_H_5_O to produce the byproduct acrolein and water. The activation of propylene governs the rate‐limiting step in the formation pathway of both PO and acrolein. At 500°C, the overall Gibbs free energy barrier (134 kJ/mol) is significantly lower than that of acrolein (208 kJ/mol). In summary, the approach of precise, atomic‐level incorporation of transition metal ions into the zeolite matrix demonstrates its viability for propene epoxidation.

## 4 CATALYSTS FOR PROPENE EPOXIDATION

**FIGURE 13:**
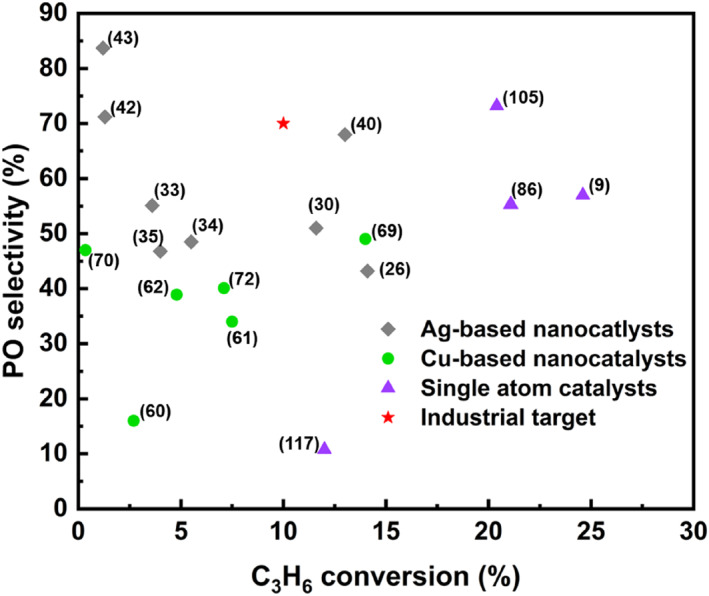
Propylene oxide (PO) selectivity and propylene conversion of various catalysts.

Researchers have achieved significant advancements in catalyst modification by employing various strategies, including altering support materials, introducing promoters, controlling crystal planes, exploring multi‐metal combinations, and designing single‐atom catalysts. Table 3 summarizes the catalytic performance of notable catalysts in recent years, while Figure 13 shows how current research findings compare with industrial benchmarks for direct propene epoxidation. Emerging single‐atom catalysts appear particularly promising for direct propene epoxidation due to their unique coordination modes and electronic structures.

**TABLE 3:** Performance and reaction conditions of various catalysts.

Catalysts	Conv. %	Selec. %	T/°C	P/MPa	Method of preparation	Gas feed composition	SV/h^−1^	Amount of catalyst	References
AgMo/Ti‐HMS_10_	14.1	43.2	400	0.1	Impregnation	22.7% C_3_H_6_:9.0%O_2_:68.3%N_2_	7500	0.1 g	[27]
Ag nanocube/La_2_O_3_	11.6	51	270	0.1	Wet impregnation	3.33% C_3_H_6_:1.67%O_2_:95%He	/	0.1 g	[25]
Ag‐Cu/BaCO_3_	3.6	55.1	200	0.1	Surfactant‐protected colloidal method	20.0% C_3_H_6_:10.0%O_2_:70.0%N_2_	2000	0.6 g	[33]
Ag_8_Cu_1_/Cs_2_O/*α*‐Al_2_O_3_	5.5	48.5	160	0.1	/	20.0% C_3_H_6_:10.0%O_2_:70.0%N_2_	2000	0.6 g	[36]
20%Ag‐0.1%Y_2_O_3_‐0.1%K_2_O/α‐Al_2_O_3_	4	46.8	245	0.1	/	20.0% C_3_H_6_:8.0%O_2_:72.0%N_2_	2000	0.5 mL	[34]
Ag‐Mo‐W/ZrO_2(pH10)_	13	68	460	0.1	Precipitation	16.67% C_3_H_6_:8.33%O_2_:75.0%N_2_	/	0.3 g	[40]
Ag‐CuCl_2_/BaCO_3_	1.3	71.2	200	0.1	Reduction‐deposition‐impregnation	20.0% C_3_H_6_:10.0%O_2_:70.0%N_2_	3000	0.6 g	[42]
Ag‐Cu‐Cl/BaCO_3_	1.2	83.7	200	0.1	Reduction‐deposition‐impregnation	20.0% C_3_H_6_:10.0%O_2_:70.0%N_2_	3000	0.6 g	[43]
VO_*x*_‐Cu	2.7	16	230	0.1	Co‐precipitation	/	/	0.2 g	[60]
Cs^+^‐CuO_*x*_/SiO_2_	7.5	34	250	0.1	Sol‐gel	/	/	0.2 g	[61]
Cu‐OH‐Cl‐TiO_2_	4.8	38.9	227	0.1	Slurry impregnation	10.0% C_3_H_6_:10.0%O_2_:80.0%N_2_	4000	0.9 g	[62]
RuO_2_‐CuO‐NaCl/SiO_2_	14	49	250	0.1	Co‐impregnation	1.0% C_3_H_6_:4.0%O_2_:95.0%He	20,000	0.005 g	[73]
RuO_2_‐CuO‐TeO_2_/SiO_2_	0.35	47	269	0.1	Co‐impregnation	2.0% C_3_H_6_:8.0%O_2_:90.0%He	152,727	0.0015 g	[68]
RuO_2_‐CuO‐Cs_2_O‐TiO_2_/SiO_2_	7.1	40.1	250	0.1	Co‐impregnation	1.0% C_3_H_6_:2.0%O_2_:97.0%He	848	0.0015 g	[70]
MoO_3_‐Bi_2_SiO_5_/SiO_2_	21.99	55.14	400	0.15	Impregnation	4.0% C_3_H_6_:16.0%O_2_:80.0%N_2_	/	0.1 g	[86]
MoO_3_/Bi_*x*_Si_1−*x*_O_2_	20.4	73.2	400	0.15	Impregnation	8.0% C_3_H_6_:16.0%O_2_:76.0%N_2_	/	0.1 g	[105]
LaCo_0.8_Co_0.2_O_3−*δ*_	12	10.8	250	0.1	Sol‐gel	10.0% C_3_H_6_:5.0%O_2_:85.0%N_2_	/	0.1 g	[117]
Co@Y	24.6	57	500	0.1	In situ hydrothermal route	4.0% C_3_H_6_:3.2%O_2_:92.8%N_2_	18,000	0.2 g	[9]

## 5 CONCLUSION

In summary, the epoxidation of propylene to PO using oxygen as the sole oxidant offers several advantages, including a straightforward process, environmental compatibility, and high atom efficiency. The current research findings highlight the significant influence of catalyst nanoparticle shape and size, as well as the electronic properties of active sites, on catalytic performance. Bimetallic or multi‐metallic catalysts often exhibit distinct electronic properties. As a result, the following directions for advancing propene epoxidation catalysts can be outlined. First, in catalyst synthesis, the selection of appropriate supports and the combination of transition metals such as Co, Ni, Cu, Ag, Mo, and Bi can be harnessed to fine‐tune the optimal catalyst composition with the desired electronic structure, ultimately enhancing PO selectivity. Concurrently, catalysts can be enhanced through the addition of promoters, such as oxides, alkali (earth) metals, halogens, among others. The addition of trace amounts of promoters has the potential to modulate the electronic properties of active sites or foster synergistic interactions between the active component and the support. Moreover, the catalyst synthesis method plays a pivotal role in its activity, necessitating the development of innovative synthesis strategies to prevent active component agglomeration and improve dispersion. There is a need to explore catalysts that maximize the exposure of the most selective facets, thereby allowing precise adjustment of the microscopic composition and atomic‐level structure of active centers. Furthermore, while research on single‐atom catalysts for propene epoxidation using oxygen is in its early stages, these catalysts have already exhibited remarkable performance. It is well known that when the size of active metals can be tuned to nanoparticles or cluster, or even to single‐atom levels to provide different metal dispersity. However, when metals change in size, their coordination environments, valence states and geometrical configurations also change, and the electronic structure will undergo a dramatic change from continuous energy bands to discrete energy levels, and ultimately to quantum confinement effect, which will directly affect the chemisorption and reactivity. In addition, the high homogeneity of single‐atom active sites facilitates the restriction of the adsorption configuration of the substrate, which leads to a highly homogeneous activation process of the reactant molecules. Therefore, SACs provide an ideal platform for elucidating the relationship between structure and function at the molecular level. Additionally, the strong coordination between the active metal and the support or promoter effectively prevents catalyst loss, offering a promising avenue for the advancement of propene epoxidation processes.

## CONFLICT OF INTEREST STATEMENT

The authors declare that they have no conflict of interest.

## Data Availability

The data that support the findings of this study are available from the corresponding author upon reasonable request.
